# Improving care for cognitive impairment in schizophrenia: current perspectives and opportunities in Portugal (a consensus development conference)

**DOI:** 10.3389/fpsyt.2026.1793913

**Published:** 2026-05-11

**Authors:** Nuno Madeira, João M. Bessa, Joaquina Castelão, Miguel Constante, Miguel Durães, Ana Rosário Fonseca, Pedro Freire, Carmen Paus, Ana Maria Rebelo, Patricia Rodrigues, Celeste Silveira, Frederico Simões do Couto

**Affiliations:** 1 Unidade Local de Saúde de Coimbra, Psychiatry Department; Coimbra Institute for Biomedical Imaging and Translational Research (CIBIT), University of Coimbra, Coimbra, Portugal; 2 School of Medicine, Life and Health Sciences Research Institute (ICVS), University of Minho, Braga, Portugal; 3 FamiliarMente - Federação Portuguesa das Associações das Famílias de Pessoas com Experiência de Doença Mental, Lisboa, Portugal; 4 Unidade Local de Saúde de Loures-Odivelas, Psychiatry and Mental Health Department, Loures, Portugal; 5 Recovery IPSS, Barcelos, Portugal; 6 Unidade Local de Saúde da Região de Leiria, Psychiatry Department, Leiria, Portugal; 7 Hospital do Mar Cuidados Especializados, Lisboa, Portugal; 8 BKC Media, Nijmegen, Netherlands; 9 Unidade Local de Saúde de Santa Maria, Psychiatry and Mental Health Department, Lisboa, Portugal; 10 Healthwords, Porto, Portugal; 11 Faculty of Medicine, University of Oporto, Porto, Portugal; Unidade Local de Saúde de São João, Psychiatry and Mental Health Department, Porto, Portugal; 12 Unidade Local de Saúde de Santa Maria, Psychiatry and Mental Health Department; Católica Medical School, Universidade Católica Portuguesa, Lisboa, Portugal

**Keywords:** cognition, cognitive assessment, cognitive impairment, cognitive remediation therapy, psychosocial functioning, schizophrenia

## Abstract

**Background:**

Cognitive impairment is a key contributor to the functional disability seen in schizophrenia, yet it remains one of the most underdiagnosed and poorly managed aspects of the disorder. In Portugal, schizophrenia imposes a significant socio-economic burden, with notable gaps in the assessment and treatment of cognitive impairment.

**Method:**

A panel of Portuguese healthcare professionals, including six psychiatrists with clinical and academic expertise, a neuropsychologist, a psychiatric nurse, and two representatives from patient associations, reviewed current evidence and practices for managing cognitive impairment in schizophrenia. The panel addressed three primary clinical questions using a consensus development conference methodology (1): What is the current state of CIAS (cognitive impairment associated with schizophrenia) assessment and management in Portugal? (2) What are the major barriers to optimal care? (3) What recommendations can improve CIAS care within the Portuguese healthcare system?

**Results:**

Several challenges hinder the routine assessment and treatment of cognitive impairment in Portugal, including the lack of validated assessment tools, the absence of national guidelines, insufficient trained healthcare professionals, and limited access to cognitive remediation therapy (CRT). Key opportunities that could improve care include establishing national guidelines for cognitive assessment and management, using validated and practical assessment tools, training healthcare professionals, integrating CRT into standard care pathways, engaging caregivers, and strengthening collaboration between healthcare institutions.

**Conclusion:**

Despite significant challenges, the Portuguese healthcare system has numerous opportunities to improve the management of cognitive impairment in schizophrenia. Critical unresolved issues include the need for additional validated Portuguese assessment instruments and the establishment of cost-effectiveness data for CRT implementation in the Portuguese context.

## 1 Introduction

Schizophrenia is a chronic, debilitating mental disorder that can have devastating effects on patients and their caregivers (1, 2). Its symptoms are typically classified into three categories: positive symptoms like hallucinations and delusions; negative symptoms such as lack of motivation and affective flattening; and cognitive deficits (2).

Cognitive impairment associated with schizophrenia (CIAS) is a relatively recent term referring to persistent deficits across multiple neurocognitive domains, including processing speed, attention, working memory, verbal and visual learning, reasoning and problem-solving, and social cognition (3, 4). These deficits are often observable before the onset of psychotic symptoms, remaining relatively stable throughout illness course (3, 5). CIAS is distinct from the cognitive decline observed in neurodegenerative conditions such as Alzheimer’s disease, being, among others, non-progressive and probably linked to neurodevelopmental changes.

Cognitive impairments have been recognized as a central aspect of schizophrenia since the late 19th century, when Emil Kraepelin introduced the concept of “dementia praecox” (6, 7). In Eugene Bleuler’s seminal works on schizophrenia, nearly a century ago, it was clear that he had a perceptive understanding of cognitive impairment as a core part of this psychotic disorder; that was consubstantiated in the definition of his fundamental symptoms—substantially cognitive in their essence, yet distinguished from what was observed in organic dementias (8). While this early understanding lost traction during the mid-20th century in favor of psychodynamic theories, interest resurged in addressing cognitive dysfunction in recent decades (9, 10). This renewed interest was probably driven by emerging neurobiological evidence, the growing recognition of CIAS as a key determinant of functional recovery, and the identification of potential treatment strategies (7, 10, 11).

Studies suggest that cognitive impairment affects over 80% of individuals diagnosed with schizophrenia (3–5, 12). Research has demonstrated a direct correlation between the severity of cognitive impairment and real-world functioning, with greater impairment often leading to unemployment, lower income, social isolation, and a higher risk of hospitalization (13–17). Caregivers also experience substantial stress, as they have to manage both the emotional and practical burdens associated with the disorder (2).

Evidence suggests that the symptoms of cognitive impairment arise from complex neurobiological mechanisms. Dysregulation of dopamine, glutamate, and cholinergic systems disrupts key circuits, particularly in the prefrontal cortex, which governs planning and social cognition (10). Structural changes like gray matter reduction, functional abnormalities, and impaired network connectivity further link these deficits to functional outcomes (18–20).

Despite its impact, CIAS remains a substantially unmet clinical need (21). The lack of routine cognitive assessments in clinical practice and the absence of guidelines on a standardized approach make it difficult to identify specific cognitive challenges accurately (21, 22). Evidence indicates that although symptomatic remission is a good indicator of better clinical status and social functioning, it does not reflect cognitive functioning (11). The lack of adequate assessment limits the adoption of targeted interventions, leaving many patients without adequate support for their cognitive needs. Additionally, antipsychotic medications, while effective in controlling positive symptoms, offer limited benefit in ameliorating cognitive deficits (23, 24). Non-pharmaceutical therapies include cognitive remediation therapy (CRT), physical exercise, and psychosocial interventions (25).

To our knowledge, there is a paucity of published data on the diagnosis and, in particular, the management of CIAS in clinical practice in Portugal. A study addressing the burden of schizophrenia on the Portuguese population in 2015 highlighted substantial socio-economic costs, suggesting significant opportunities for improvement within the healthcare system (26). Currently, there are no established national guidelines for the diagnosis and management of CIAS, and structured cognitive assessment and intervention remain rare in clinical practice. In addition to the absence of guidelines, it is anticipated that other challenges, such as limited availability of validated assessment tools, insufficient training of mental health professionals, and restricted access to CRT, and other treatments could also hinder optimal care delivery.

To address these gaps, a multidisciplinary panel of Portuguese experts was convened to (1) offer a perspective on CIAS assessment and management (2), understand the major challenges, and (3) formulate context-specific recommendations aimed at improving care within the national healthcare system.

A consensus development conference was set, following the ACCORD guidelines for consensus methodology (26). This methodology was chosen because the published evidence on CIAS is poor and there is a need to achieve synthesis of ideas from different stakeholders to identify future directions, consistent with the main indication for this methodology. The ACCORD checklist is provided in a **Supplementary S1 file**.

Although the practical aims are mainly targeted for a national audience, it is anticipated that the synthetized data will be helpful to other countries to have comparative data and/or have a baseline expected data to work on. The study was not registered.

The project was led by the chair, who wrote the draft project, chose the panel members, chaired the discussion, and was responsible for the overall organization of the study.

The panel intended to be multidisciplinary, including the most relevant stakeholders, from academic and regional health centers, private, public, and social (psychiatrists neuropsychologists, patients advice groups, and nurses). In spite of being a small country, a geographical distribution was tried. The criteria were psychiatrists with recognized clinical and/or academic expertise in schizophrenia, neuropsychologists specialized in cognitive assessment and rehabilitation of patients with schizophrenia, psychiatric nurses with experience in schizophrenia care, and patient and caregiver representatives from organizations that in Portugal provide assistance and advocacy to mental health patients and caregivers. The invitation was personal, and panelists were allowed to suggest other members of the panel.

To prepare for the meeting, CIAS guidelines have been sent to the panelists. A non-systematic review was conducted on electronic databases such as PubMed. Key terms for the search were “schizophrenia” and (“cognition” or “cognitive performance” or “CIAS” or “cognitive impairment”) and “guidelines” up to 2024. Additional guidelines were retrieved from other publications. A short presentation was made in the early part of the conference to ensure a common background and contextual information.

The meeting followed the consensus development conference methodology (27, 28). Three main questions were presented (1): what is your perspective on CIAS assessment and management in Portugal? (2) what is your perspective on the major challenges for CIAS assessment and management in Portugal? and (3) formulate context-specific recommendations, aimed at improving CIAS assessment and management in Portugal, within the national healthcare system.

Each question was presented and discussed, and each panelist freely shared their own perspectives. The chair ensured that every participant was included and engaged in the discussion. These different perspectives were written, a summary was made, and a conclusion was reached by consensus, and provisionally approved. To increase scientific accuracy, after the meeting, conclusions were written and sent to the panelists for approval, answering accordingly to a five-point Likert scale (Strongly Disagree, Disagree, Neutral, Agree, or Strongly Agree). The statement was approved if more than 75% of the responses were Agree or Strongly Agree. Results are expressed in % of approval, mean ± SD, and % valid responses.

This paper outlines their conclusions and proposed strategies to improve treatment and support for individuals with schizophrenia in Portugal.

## 2 Perspectives

### 2.1 Assessment of CIAS

#### 2.1.1 Clinical question: Are patients with schizophrenia in Portugal systematically assessed for cognitive impairment using validated instruments?

The baseline information was the European Psychiatric Association guideline on schizophrenia, which strongly advocates for the assessment and management of cognitive impairment and negative symptoms as integral components of standard care (21). This guideline highlights the importance of cognitive assessments in guiding clinicians to effectively select and adapt pharmacological and behavioral treatments. Despite this emphasis, cognitive impairment often goes unrecognized, primarily due to the absence of standardized assessment protocols, leaving many deficits undetected by commonly used rating scales (22). As a result, patients with cognitive deficits may receive insufficient follow-up and support.

The experts agreed that, in Portugal, patients with schizophrenia have relatively good access to care; however, assessment of cognitive impairment remains inconsistent and varies significantly between institutions. There are 2 validated tools for European Portuguese [Brief Cognitive Assessment Tool for Schizophrenia (B-CATS) and Screen for Cognitive Impairment in Psychiatry (SCIP)] (29, 30). At some institutions, clinicians use neurocognitive assessment instruments like the Trail Making Test or the Wisconsin Card Sorting Test, but these are not validated for schizophrenia-specific impairments. In contrast, the situation seems more structured in the context of first-episode psychosis (FEP), where several hospitals have implemented protocols for cognitive assessment. These programs are already available for cognitive evaluation in FEP (31, 32).

### 2.2 Management of CIAS

#### 2.2.1 Clinical question: Are evidence-based interventions for cognitive impairment (both pharmacological and non-pharmacological) routinely implemented in Portuguese psychiatric care?

##### 2.2.1.1 Non-pharmacological strategies

CRT, physical exercise, and psychosocial interventions are among the strategies shown to improve cognitive function (33, 34).

In general, in Portugal, non-pharmacological approaches to manage cognitive impairment in schizophrenia remain underdeveloped, but several interventions hold promise based on international evidence.

###### 2.2.1.1.1 Cognitive remediation therapy

CRT is the most well-established non-pharmacological intervention for cognitive impairment associated with schizophrenia. This evidence-based therapy involves structured, goal-oriented exercises designed to enhance cognitive domains such as memory, attention, executive function, and social cognition (33). A meta-analysis of 24 trials (1,262 participants) involving patients receiving computerized cognitive drill and practice training reported small to moderate effect sizes across cognitive domains, showing significantly more improvement on attention (ES = 0.31, *p* = 0.001) and working memory (ES = 0.38, *p* < 0.001). Marginally significant effect sizes were found for other domains (34). A more recent meta-analysis (130 studies, 8,851 participants) specifically on CR found significant effects on global cognition (*d* = 0.29, 95% CI 0.24–0.34) and global functioning (*d* = 0.22, 95% CI 0.16–0.29). Key moderators of enhanced response included the presence of an active trained therapist and structured development of cognitive strategies, among others (33).

In Portugal, CRT is offered in a limited capacity and primarily in specialized settings, such as day hospitals, and is inconsistently implemented throughout the country. Home-delivered interventions have been explored in FEP cohorts and other psychotic disorders (35, 36).

###### 2.2.1.1.2 Other interventions

For physical exercise, three meta-analyses have found an improvement in global cognition, although the domains improved were different. A first meta-analysis (10 trials, 385 patients) demonstrated that exercise significantly improved global cognition (*g* = 0.33, 95% CI 0.13–0.53), with indication that greater amounts of exercise and interventions that were supervised by physical activity professionals are associated with larger improvements. Randomized controlled trials showed even larger effects (*g* = 0.43). Specific cognitive domains showed improvements in working memory, social cognition, and attention/vigilance (37). An updated meta-analysis (15 studies) confirmed improvements in global cognition (SMD = 0.21), although differences in the domains were found (attention/vigilance, working memory, and verbal learning (38). A 2024 meta-analysis (22 studies, 1,066 patients) found even larger effects on global cognition (SMD = 0.73, 95% CI 0.46–1.00), with aerobic exercise superior to resistance and mindfulness exercise (SMD = 0.76) (39).

Psychosocial interventions, including skills training and social cognition training, are essential for reinforcing cognitive improvements. These approaches target practical applications of cognitive skills, such as managing daily tasks and navigating social interactions, often impaired in schizophrenia (40).

The panel considered that the availability of these interventions varies widely between institutions in Portugal. Some hospitals provide cognitive training programs, but these are not uniformly available, and the lack of standardized practices limits their effectiveness.

Psychoeducation for caregivers can improve understanding of cognitive deficits and enable them to support patients more effectively (41, 42).

However, caregiver-focused programs are not widely accessible in Portugal, which may impact patient adherence to interventions.

##### 2.2.1.2 Pharmacological strategies

Pharmacological interventions in Portugal, as in other countries, primarily focus on maintaining clinical stability through antipsychotics (25). While medication effectively reduces positive symptoms, the impact on cognitive deficits is minimal (23).

###### 2.2.1.2.1 Second-generation antipsychotics

Second-generation antipsychotics (SGAs) are preferred over first-generation agents due to their comparatively favorable cognitive profiles. International evidence suggests that medications such as clozapine, olanzapine, and risperidone may provide modest improvements in specific cognitive domains (23). However, in Portugal, the use of SGAs is not explicitly aligned with cognitive outcomes, and clinicians often prioritize their use for managing psychosis rather than targeting cognitive impairment (43).

###### 2.2.1.2.2 Emerging pharmacological strategies

The potential of agents targeting glutamatergic pathways, such as N-acetylcysteine, as well as dopaminergic enhancers and NMDA receptor modulators (44, 45), has been explored as therapeutic tools for CIAS.

The panel considered that the use of these new agents in Portugal is very limited.

###### 2.2.1.2.3 Rational pharmacological simplification

Another strategy involves the careful reduction of medications that negatively impact cognitive function, particularly in stabilized patients. Gradual de-escalation of antipsychotic dosage has shown some favorable outcomes (46, 47). Beyond antipsychotics, reducing or discontinuing medications with significant anticholinergic properties—e.g., drugs used for extrapyramidal side effects—can improve cognition, highlighting the cumulative negative effect of anticholinergic load on multiple cognitive domains and emphasizing the risks of polypharmacy. (48, 49) Taken together, available evidence supports a tailored, multidisciplinary pharmacological approach that includes dose minimization, anticholinergic load reduction, and rational polypharmacy as feasible strategies to preserve and enhance cognitive function in individuals with schizophrenia.

The panel acknowledged uncertainty regarding current polypharmacy practices in Portugal. While polypharmacy was prevalent several years ago, its specific contribution to CIAS was not systematically assessed (43). The panel believes that polypharmacy rates, including anticholinergic medication use, have declined; nevertheless, these practices may still adversely affect CIAS.

#### 2.2.2 Panel consensus on the current state of CIAS care in Portugal

Concerning the assessment and management of CIAS in Portugal, the experts have agreed that:

1. Assessment
1.1. The assessment of cognitive impairment remains inconsistent and varies significantly between institutions (100% approved, mean 4.70 ± 0.48; 100% valid responses).
1.2. Institutions appear to use both CIAS-specific instruments and general cognitive assessment tools (100% approved, mean 4.20 ± 0.42; 100% valid responses).
1.3. Both validated (B-CATS and SCIP) and non-validated instruments seem to be used in CIAS assessment across institutions (100% approved, mean 4.33 ± 0.50; 90% valid responses).
1.4. For FEP, CIAS assessment seems more standardized (100% approved, mean 4.67 ± 0.50; 90% valid responses).
2. Non-pharmacological approaches
2.1. Non-pharmacological approaches are usually offered in a limited capacity and primarily in specialized settings and is inconsistently implemented throughout the country (100% approved, mean 4.70 ± 0.48; 100% valid responses).
2.2. Non-pharmacological programs differ between different centers (100% approved, mean 4.80 ± 0.42; 100% valid responses).
3. Pharmacological
3.1. Current pharmacological treatment in Portugal primarily targets psychotic symptoms rather than cognitive impairment (100% approved, mean 4.60 ± 0.52; 100% valid responses).
3.2. Novel pharmacological approaches for cognitive enhancement in schizophrenia remain largely unexplored in Portugal (77.8% approved, mean 4.44 ± 0.88; 90% valid responses).
3.3. Although there are clear indications of improvement, polypharmacy and anticholinergic burden may still constitute potential concerns in prescribing practices in Portugal, concerning CIAS impact (90% approved, mean 4.30 ± 0.67; 90% valid responses).

### 2.3 Challenges in managing cognitive impairment

#### 2.3.1 Systemic question: What structural, educational, and resource-related obstacles prevent the implementation of evidence-based CIAS assessment and treatment in Portugal?

The main challenges identified for managing CIAS are the lack of trained mental healthcare practitioners to recognize cognitive impairment, the existence of feasible and acceptable screening and treatment planning protocols, and the insufficient perceived value of cognitive interventions (21, 22, 33).

A primary challenge in Portugal is the fragmented approach to cognitive assessment and intervention. The panel considered that cognitive impairment is probably underrecognized in clinical settings due to a lack of standardized screening protocols and validated tools adapted for the Portuguese population. While some hospitals have adopted assessment methods such as the B-CATS or adapted neurocognitive assessment tools, these are neither widely used nor systematically applied across institutions.

**Figure 1:**
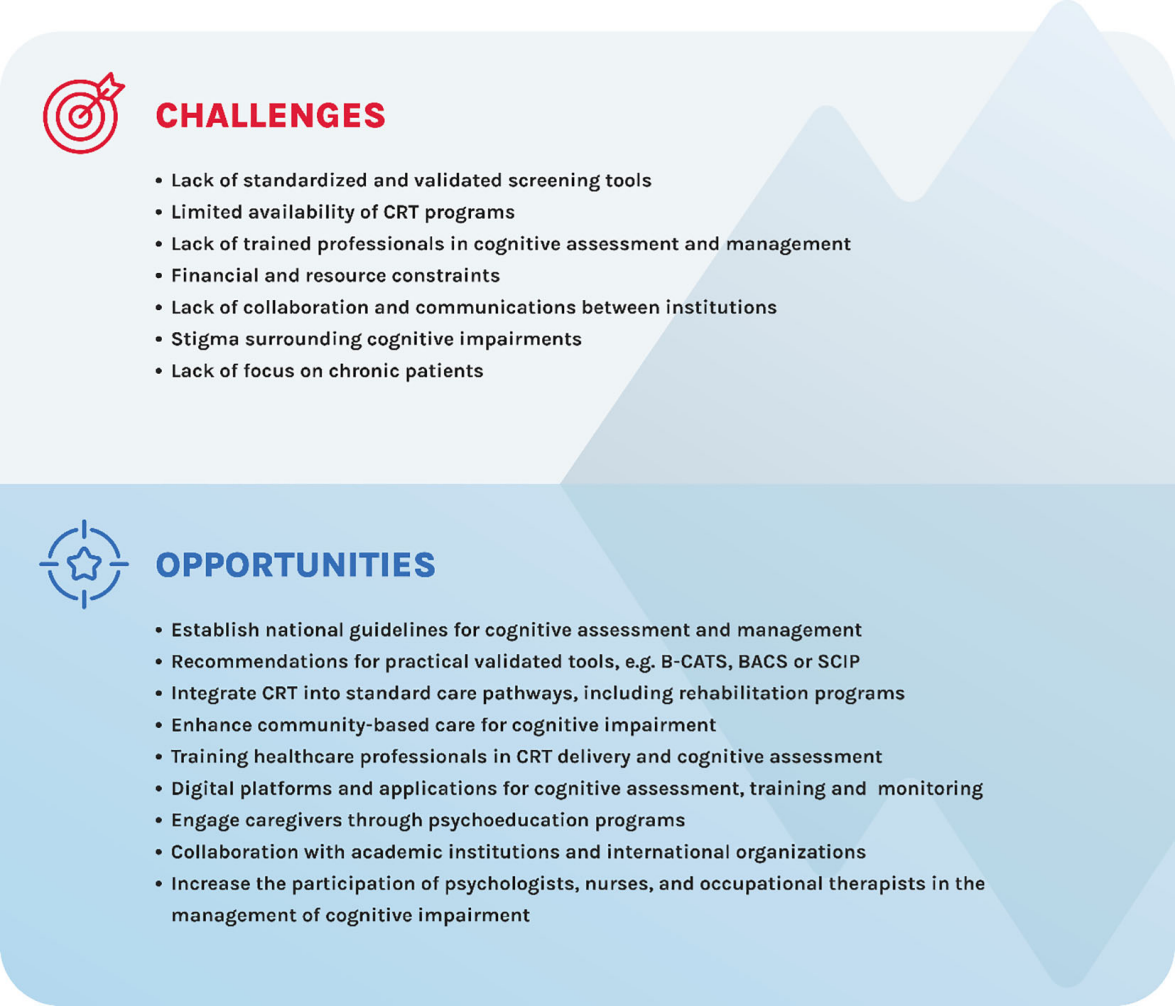
Challenges in managing cognitive impairment in schizophrenia and opportunities for improving care in the Portuguese healthcare system.

Experts highlighted several challenges to integrate cognitive assessments into routine care. These include limited availability of CRT programs, lack of standardized protocols, shortage of healthcare professionals trained on available assessment tools, and the lack of resources to administer tests, particularly the limited availability of psychologists, nurses, and occupational therapists. Some healthcare professionals have limited awareness of the functional implications of cognitive deficits, and others lack hope in improving symptoms. Two additional issues were discussed: the collaboration between hospital’s mental health teams is generally low, and it is not clear if there are differences among the awareness of the functional implications of cognitive deficits among mental health teams. Financial and time constraints, besides limited infrastructures, further hinder progress. Hospitals and mental health services often lack the resources to implement evidence-based cognitive interventions on a broader scale. Additionally, collaboration and communication between institutions are sparse, leading to a lack of continuity and shared learning across the healthcare system. Stigma surrounding cognitive impairments, both among healthcare providers and the general population, further contributes to underdiagnosis and suboptimal management of cognitive deficits. For example, the lack of focus on chronic patients, reinforced by the mistaken belief that improvement in the chronic phase is residual, has resulted in an inadequate assessment and treatment of cognitive impairment in this group (**Figure 1**).

Another significant challenge lies in the limited availability of validated assessment tools adapted to the Portuguese population. For example, while the B-CATS and the SCIP have been adapted for use in Portugal, their application remains sparse, and many clinicians are not aware of their existence (29, 30).

#### 2.3.2 Panel consensus on barriers to optimal CIAS care

Concerning the main challenges for the assessment and management of CIAS in Portugal, the experts have agreed that:

1. The cognitive assessment practices are fragmented and vary across institutions (88.89% approved, mean 4.44 ± 0.73; 90% valid responses).
2. Standardized screening protocols are insufficient (90% approved, mean 4.50 ± 0.97; 100% valid responses).
3. The systematic use of validated assessment tools is limited (100% approved, mean 4.40 ± 0.52; 100% valid responses).
4. There is a paucity of validated instruments for CIAS assessment in European Portuguese (100% approved, mean 4.30 ± 0.48; 100% valid responses).
5. The resources and trained professionals to implement cognitive assessment and interventions are limited (100% approved, mean 4.52 ± 0.48; 100% valid responses).
6. The awareness and limited prioritization of cognitive impairment in clinical practice are globally low (90% approved, mean 4.40 ± 0.97; 100% valid responses).
7. The coordination and dissemination of tools and practices across institutions is insufficient (90% approved, mean 4.70 ± 0.48; 100% valid responses).

### 2.4 Opportunities for improvement/recommendations

#### 2.4.1 Implementation question: What actionable recommendations, graded by strength and feasibility, can bridge the gap between current practice and evidence-based CIAS care?

Despite these challenges, the Portuguese healthcare system has several avenues to enhance the management of cognitive impairment in schizophrenia (**Figure 1**). Establishing national guidelines for cognitive assessment and treatment is a critical first step. These guidelines should advocate for the systematic use of validated tools, such as the B-CATS and SCIP, which offer practical options for routine clinical use.

International available tools such as the Measurement and Treatment Research to Improve Cognition in Schizophrenia (MATRICS) Consensus Cognitive Battery (MCCB) are considered gold standards for assessing cognitive domains, including processing speed, working memory, and social cognition (50). While highly comprehensive, the MCCB is time-intensive, requiring up to 90 min to administer, which limits its use in routine clinical settings (50, 51). Alternative tools, like the B-CATS, Brief Assessment of Cognition in Schizophrenia (BACS), and the SCIP, offer more practical options, requiring significantly less time to complete (approximately 10, 35, and 15 min, respectively) (52–54). These tools are well-suited for initial screenings and could help streamline the identification of cognitive impairment in Portuguese mental health services. Incorporating digital cognitive tools, which have shown promise in improving accessibility and efficiency in other contexts, could also be a cost-effective and easy-to-administer solution for routine evaluations.

To implement a systematic assessment of cognitive functions, FEP programs could also be leveraged. In fact, in some Portuguese hospitals, these programs are already available for cognitive evaluation (35, 36). The panel considered this an example that could be expanded to other hospitals.

Raising awareness among healthcare providers and policymakers about the importance of assessing cognitive impairments, including on chronic patients, was also proposed. Cognitive deficits are strongly correlated with real-world functioning, influencing employment, social relationships, and overall quality of life (13–17). By prioritizing cognitive assessments and integrating them into standard care, Portuguese mental health services can better address this unmet need and ultimately improve outcomes for patients with schizophrenia.

Expanding access to CRT by integrating it into standard care pathways can also significantly improve outcomes (55, 56). Training healthcare professionals, including psychiatrists, psychologists, neuropsychologists, nurses, and occupational therapists, in CRT delivery and cognitive assessment is essential to address the current lack of knowledge about CIAS. Incorporating CRT within existing psychosocial rehabilitation programs and community-based care can ensure that these interventions reach more patients. While community-based approaches are gradually developing in Portugal, there are insufficient data on their effectiveness. Further efforts are needed to enhance implementation and impact.

Engaging caregivers through structured psychoeducation programs was also found to be important. Equipping caregivers with the knowledge and skills to support cognitive rehabilitation can enhance intervention adherence and provide patients with greater daily support.

Collaboration with academic institutions and international organizations could drive innovation and foster the development of evidence-based interventions tailored to the healthcare in Portugal. Increased funding and policy support would also strengthen infrastructure, develop specialized services, and integrate cognitive care into broader mental health strategies. A survey would also provide real data to have a more accurate picture on the Portuguese reality.

Also, efforts to participate in international studies or conduct local research could pave the way for introducing innovative treatments.

By addressing these challenges and seizing available opportunities, the Portuguese healthcare system can significantly improve the identification and management of cognitive impairments in schizophrenia, leading to better functional outcomes and quality of life for patients.

#### 2.4.2 Panel consensus recommendations with strength grading

Concerning the main Opportunities for Improvement and Recommendations of CIAS in Portugal, the experts have agreed to recommend to:

Recommendation 1: Increase the availability of validated cognitive assessment tools for the Portuguese population (100% approved, mean 4.75 ± 0.46; 80% valid responses). (Strong recommendation; High priority);

Recommendation 2: Implement routine cognitive screening using brief and feasible instruments (100% approved, mean 4.70 ± 0.48; 100% valid responses). (Strong recommendation; High priority).

Recommendation 3: Develop national guidelines for the assessment and treatment of cognitive impairment in schizophrenia (90% approved, mean 4.7 ± 0.48; 100% valid responses). (Strong recommendation; High priority).

Recommendation 4: Expand and scale cognitive assessment through FEP programs (90% approved, mean 4.50 ± 0.71; 100% valid responses). (Moderate recommendation; Medium priority).

Recommendation 5: Expand access to CRT and strengthen professional training (90% approved, mean 4.40 ± 0.70; 100% valid responses). (100% approved, mean 4.7 ± 0.48; 100% valid responses). (Strong recommendation; High priority)

Recommendation 6: Expand and scale cognitive assessment through FEP programs (90% approved, mean 4.4 ± 0.70; 100% valid responses). (Moderate recommendation; Medium priority).

Recommendation 7: Promote research, service development, and stakeholder engagement (100% approved, mean 4.70 ± 0.48; 100% valid responses). (Moderate recommendation; Medium priority).

#### 2.4.3 Unresolved issues and knowledge gaps

Despite consensus on key recommendations, several critical issues remain unresolved and require future investigation:

1. Validation gap: Only two cognitive assessment tools (B-CATS and SCIP) are currently validated for European Portuguese. Additional instruments (MCCB, BACS, CAI, and social cognition measures) require formal validation studies.
2. Polypharmacy quantification: Current rates of polypharmacy and anticholinergic burden in Portuguese practice remain unclear. National audit data are needed to quantify the scope of this problem.
3. CRT implementation models: The optimal model for CRT delivery in Portugal (individual vs. group, computerized vs. therapist-led, and hospital-based vs. community-based) has not been determined. Cost-effectiveness data specific to the Portuguese healthcare context are lacking.
4. Resource requirements: The specific number of trained professionals, time allocation, and infrastructure investments required for widespread CIAS screening and intervention have not been systematically calculated.
5. Chronic Patient Outcomes: Evidence on the effectiveness of cognitive interventions in chronic, stable patients in Portugal is limited, despite international data suggesting benefit.
6. Inter-institutional variation: The degree of variation in CIAS assessment and management practices across Portuguese institutions has not been formally quantified through systematic survey.

## 3 Discussion

The panel reached consensus on three critical domains that reflect the substantial gap in CIAS assessment and management. The panel identified that cognitive assessment remains fragmented and inconsistent across Portuguese institutions, with validated tools (B-CATS and SCIP) available but underutilized. In addition, there is a need to increase the validated assessment instruments for European Portuguese, while non-pharmacological interventions like CRT are offered only in limited, specialized settings rather than as standard care.

These findings directly address the clinical question by documenting that despite good general access to psychiatric care in Portugal, the specific needs of patients with cognitive impairment remain largely unmet. Most of the panelists agree on most of the statements, strengthening the evidence base needed to justify healthcare system changes and cooperation among institutions. These three main findings will be discussed, along with the most recent guidelines (21, 22, 25, 57–59).

Comparing the most recent and widely used guidelines and recommendations (**Table 1**, **Supplementary File S2**), cognitive impairment is consistently recognized as a core feature of schizophrenia that requires routine attention in clinical care. Most guidelines recommend that cognition be assessed in all individuals with schizophrenia, including those in early or at-risk stages. The RANZCOP guidelines (2026) (58) take a more selective approach by limiting assessment to children, to those with developmental difficulties, and to older adults with suspected cognitive decline. Most recent guidelines agree that assessment should not be limited to a single time point. Instead, it should occur across the course of illness, including at diagnosis, during different stages of progression, and in response to treatment, with some guidance less explicitly recommending ongoing monitoring over time. The Italian guidelines highlight a pragmatic stance (25), noting that in resource-limited settings, any form of cognitive assessment is preferable to none. This panel recommended to increase the assessment of CIAS, in line with this programmatic approach. In the future, the development of national guidelines should indicate, in the context of low resources, which patients, and when, should be assessed for CIAS. In this way, one of the main conclusions of the present study—the actual fragmented approach to CIAS assessment—is addressed.

**Table 1:** Comparison of different guidelines regarding cognitive impairment in schizophrenia.

	European Psychiatric Association guidelines (21, 25)	Italian guidelines (22)	American Psychiatric Association Guidelines (57)	Australian and New Zealand Journal of Psychiatry guidelines (58)	VA/DoD Guideline (FEP) (59)
Assessment					
Who?	In all patients with schizophrenia, including CHR	In all patients with schizophrenia, including CHR	Patients with schizophrenia	Child with schizophrenia or a history of significant developmental delay or intellectual disability. Older adult with schizophrenia and a history of significant cognitive decline.	Not specifically stated, but seems to apply to all FEP
When?	All phases of the disorder	Different stages of the illness, at the start, and at the completion of dedicated treatment programs	At the diagnosis		Should be monitored
How?	The six neurocognitive domains identified by the MATRICS initiative should be carefully assessed in subjects with schizophrenia	In the context of limited resources, providing any type of cognitive assessment is better than providing no cognitive assessment at all. CAI is freely downloadable.			
	Both self-reports and observer reports of cognitive ability	Both self-reports and observer reports of cognitive ability			
	MATRICS and BACS	MCCB represents the most appropriate and complete validated tool currently. BACS could be used as an alternative.			
	SCIP for sreening	SCIP for sreening			
	SCoRS or CAI can be used as co-primary measures	Interview-based instruments, such as SCoRS and CAI			
	BLERT (emotional processing), and Hinting task (ToM) and TASIT (emotional processing and ToM)	BLERT (emotional processing), and Hinting task (ToM) and TASIT (emotional processing and ToM)			
	RAD, MiniPONS, and SAT-MC should not be used to assess social perception. AIHQ and the Trustworthiness Task	For other social cognitive domains, no available test has sufficiently reliable psychometric properties to justify its recommendation.			
		Metacognition should also be considered and assessed with the MAS-A.			
Treatment CRT					
Who?	People living with schizophrenia and with cognitive impairment	People living with schizophrenia and with CIAS	Suggests that patients with schizophrenia receive cognitive remediation	Individuals with schizophrenia, if cognitive impairment is present and should be specifically offered when cognitive deficits are affecting recovery and function.	People living with schizophrenia and with cognitive deficits
When?				All stages of illness including at-risk mental states, early psychosis and in people with established illness	
How?	Should be delivered by a trained therapist and integrated in a psychosocial rehabilitation program.	Cognitive remediation interventions should be delivered by a trained and active therapist. Repeated practice of cognitive exercises, structured development of cognitive strategies, and use of techniques to improve the transfer of cognitive gains into the real-world. In a context of limited resources, a paper and pencil version of the Cognitive Remediation Therapy (CRT) can be obtained at no cost.	A number of different cognitive remediation approaches have been used (group or computer-based formats), in an effort to enhance cognitive processes such as attention, memory, executive function, social cognition, or meta-cognition Some programs have focused on improving cognitive flexibility, working memory, and planning. Meta-cognitive approaches can be used.	Cognitive remediation programs administered by clinicians specifically trained in CRT and do not suggest web-based trainings (supported by poor evidence).	Cognitive training, cognitive remediation, or both for cognitive deficits
Other interventions	Physical exercise should be integrated. Lifestyle interventions could have mild positive effects. Other psychosocial interventions may help, but current evidence is insufficient to recommend them.	Physical exercise should be integrated into rehabilitation projects. Although factors such as metabolic syndrome, smoking, poor diet, sedentary lifestyle, sleep issues, and substance use disorders were mentioned, no recommendation was made.			
Treatment pharmacology	Keep anticholinergic burden to a minimum. Benzodiazepines could have a negative impact on cognition.No systematic assessment of their impact on cognition in is currently available.	Reducing the negative impact on CIAS of anticholinergic or benzodiazepine medications, particularly frequent with first generation APDs	Not specifically recommended, but worries with anticholinergic medication		Non-specific recommendations, but dispersed concerns about the causes of cognitive impairment (e.g., Recommendation 15: “There was concern regarding the adverse cognitive effects of using topiramate”).
	Second-generation antipsychotics are recommended for their favorable cognitive profile compared to first-generation antipsychotics	SGAs are recommended for their favorable cognitive profile compared to FGAs.			
	Antidepressants (a significant positive effect was observed), but the size of the positive effect was minimal. Other drugs (with potential effect on CIAS) should be considered as off-label treatments.	Several potentially beneficial molecules are currently being developed and investigated, but the available evidence is not sufficient to recommend.			There is insufficient evidence to recommend for or against augmentation with any non-antipsychotic medication for treatment of cognitive symptoms.
	Currently, the available literature does not allow to recommend TMS as an evidence-based treatment for cognitive impairment.				

In terms of assessment methods, only the European guidelines recommend a multidimensional and standardized approach. This includes evaluating key neurocognitive domains such as attention, memory, executive functioning, and social cognition, using both objective performance-based measures and subjective reports from patients and observers. These two guidelines propose specific assessments for CIAS. Only one of the proposed assessment tools (SCIP) has been validated for use in Portugal, which clearly supports the second important finding of this study, the recommendation to expand the number of validated instruments available. The panel could have provided guidance on which instruments should be prioritized for validation; however, those recommended in the European guidelines appear to be the most appropriate candidates.

CRT is uniformly recommended as the primary intervention for CIAS. It is generally indicated for individuals who demonstrate cognitive deficits, particularly when these impairments affect functional outcomes or recovery. The timing of intervention is often not clearly specified, but it is generally understood to be applicable at all stages of the illness. Some guidelines (58) explicitly recommend its use across the full course of the condition, including early psychosis and chronic phases. There is strong consensus that CRT should be delivered by trained therapists and embedded within a broader psychosocial rehabilitation framework (21, 22, 58). Effective programs typically involve repeated cognitive exercises, structured development of cognitive strategies, and techniques aimed at transferring cognitive gains into real-world functioning. Depending on the guideline, a range of delivery formats is acceptable, including individual, group, and computer-based approaches, although purely web-based interventions are discouraged in some guidance due to limited supporting evidence. In low-resource settings, simpler formats such as paper-and-pencil interventions are considered reasonable alternatives. Other non-pharmacological interventions are acknowledged but less strongly supported. The current recommendations address these issues by advocating for the training of therapists in CRT. Future Portuguese guidelines should clarify which patients are most likely to benefit and identify the CRT models best suited to the Portuguese population and healthcare system.

Pharmacological approaches to cognitive impairment remain limited, and no medications are strongly recommended specifically for this purpose. Instead, guidelines emphasize minimizing factors that may worsen cognition, particularly reducing the use of anticholinergic medications and benzodiazepines (21, 22, 25, 57). SGAs are generally preferred over first-generation agents due to a more favorable cognitive profile. Some evidence suggests that antidepressants may have small positive effects, but these are clinically modest (25). Other somatic strategies are still under investigation, and there is insufficient evidence to support their routine use (25). The Portuguese consensus panel’s findings align with international evidence reflecting the global absence of effective procognitive agents. Also, despite growing awareness, polypharmacy and anticholinergic burden may still remain persistent concerns in Portuguese prescribing. By specifically addressing this issue, these recommendations may help to improve their use.

The under-recognition of cognitive impairment in schizophrenia, the limited implementation of CRT, and the preoccupation with anti-cholinergic and benzodiazepines overuse seem to reflect challenges commonly reported across international mental health system (21, 22, 25, 60). On the other hand, some of perspectives are grounded in the Portuguese context, such as the inter-institutional variability, the lack of validation protocols, and the organizational structure of FEP programs. Distinguishing between these universal and context-specific elements can be considered important to be addressed by both local policy development or broader international practice.

This paper presents the first structured national effort to assess and propose improvements in the care of CIAS in Portugal. One of its major strengths lies in its multidisciplinary panel composed of psychiatrists, psychologists, nurses, and patient advocacy representatives. This diverse composition allowed for a comprehensive and context-sensitive discussion, incorporating clinical, scientific, and lived-experience perspectives. Its methodology, while not empirical in nature, followed an iterative, consensus-based approach, combining literature review with expert reflection.

Another notable strength is the panel’s alignment with current international guidelines. By highlighting the gap between international best practices and current national implementation, the paper builds a roadmap for advancing care.

Nonetheless, this paper also has important limitations. First, as a position paper rather than a systematic review or original empirical study, findings are inherently shaped by the perspectives and experiences of the panel. Although participants were selected for their recognized expertise and representativeness, the absence of formal stakeholder consultation limits generalizability.

Despite these constraints, the value of this paper lies in its role as a foundational step. It raises awareness of an under-addressed clinical domain, identifies practical and policy-level targets for change, and sets the stage for future initiatives, including national guideline development and the validation of additional cognitive assessment instruments, which remain under-assessed in current practice.

## 4 Future recommendations

**Figure 2:**
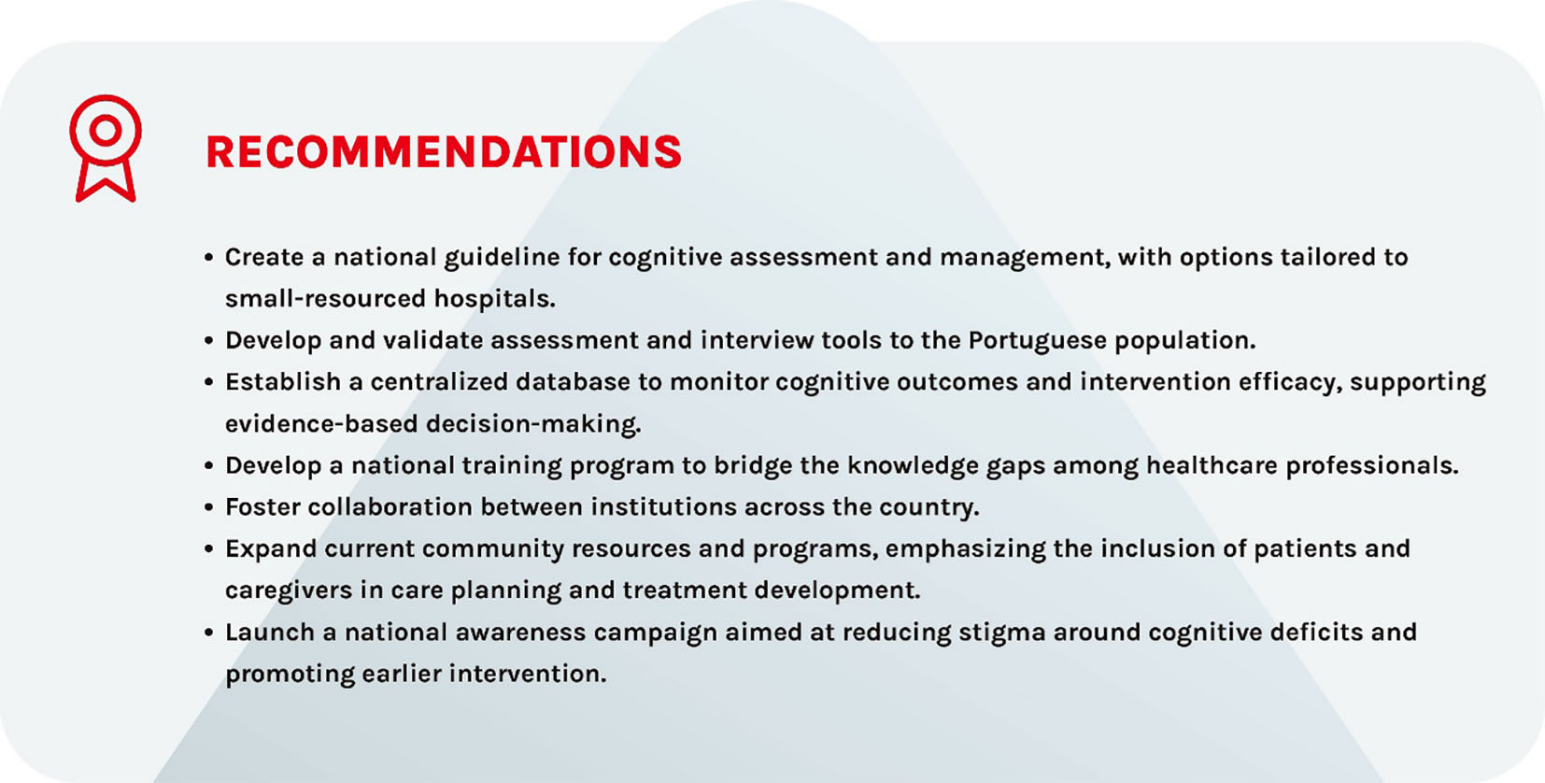
Recommendations to improve CIAS care in Portugal.

Advancing care for cognitive impairment in schizophrenia in Portugal requires a long-term, collaborative approach that integrates research, clinical practice, and policymaking (**Figure 2**). Future clinical guidelines for the management of CIAS would benefit from a systematic, evidence-based approach, using established frameworks like GRADE (60), and reflect a collaborative approach that integrates research, clinical practice, and policymaking to address current challenges and build a cohesive system of care (61, 62). By combining methodological rigor with a forward-looking, system-level vision, such guidelines can more effectively contribute to transforming care for individuals with schizophrenia in Portugal, ultimately empowering them to achieve greater independence and quality of life.

## Data Availability

The raw data supporting the conclusions of this article will be made available by the authors, without undue reservation.

## References

[1] da Motta C Pato MT Barreto Carvalho C Castilho P . The neurocognitive and functional profile of schizophrenia in a genetically homogenous European sample. Psychiatry Res. (2021) 304:114140. doi: 10.1016/j.psychres.2021.114140. PMID: 34340130

[2] Owen MJ Sawa A Mortensen PB . Schizophrenia. Lancet. (2016) 388:86–97. doi: 10.3109/10401239309148967. PMID: 26777917 PMC4940219

[3] Keefe RS Fox KH Harvey PD Cucchiaro J Siu C Loebel A . Characteristics of the MATRICS Consensus Cognitive Battery in a 29-site antipsychotic schizophrenia clinical trial. Schizophr Res. (2011) 125:161–8. doi: 10.1016/j.schres.2010.09.015. PMID: 21075600

[4] Schulz SC Murray A . Assessing cognitive impairment in patients with schizophrenia. J Clin Psychiatry. (2016) 77:3–7. doi: 10.4088/jcp.14074su1c.01. PMID: 26919051

[5] Carbon M Correll CU . Thinking and acting beyond the positive: the role of the cognitive and negative symptoms in schizophrenia. CNS Spectr. (2014) 19:38–52. doi: 10.1017/s1092852914000601. PMID: 25403863

[6] Adityanjee Aderibigbe YA Theodoridis D Vieweg VR . Dementia praecox to schizophrenia: the first 100 years. Psychiatry Clin Neurosci. (1999) 53:437–48. doi: 10.1046/j.1440-1819.1999.00584.x. PMID: 10498224

[7] Silveira C Marques-Teixeira J de Bastos-Leite AJ . More than one century of schizophrenia: an evolving perspective. J Nerv Ment Dis. (2012) 200:1054–7. doi: 10.1097/NMD.0b013e318275d249 23197119

[8] Madeira N Caldeira S Bajouco M Pereira AT Martins MJ Macedo A . Social cognition, negative symptoms and psychosocial functioning in schizophrenia. Clin Neurosci Ment Health. (2016) 1. doi: 10.21035/ijcnmh.2016.3.1

[9] Javitt DC . Cognitive impairment associated with schizophrenia: from pathophysiology to treatment. Annu Rev Pharmacol Toxicol. (2023) 63:119–41. doi: 10.1146/annurev-pharmtox-051921-093250. PMID: 36151052

[10] McCutcheon RA Keefe RSE McGuire PK . Cognitive impairment in schizophrenia: aetiology, pathophysiology, and treatment. Mol Psychiatry. (2023) 28:1902–18. doi: 10.1038/s41380-023-01949-9. PMID: 36690793 PMC10575791

[11] Brissos S Dias VV Balanzá-Martinez V Carita AI Figueira ML . Symptomatic remission in schizophrenia patients: relationship with social functioning, quality of life, and neurocognitive performance. Schizophr Res. (2011) 129:133–6. doi: 10.1016/j.schres.2011.04.001. PMID: 21514793

[12] Keefe RS Eesley CE Poe MP . Defining a cognitive function decrement in schizophrenia. Biol Psychiatry. (2005) 57:688–91. doi: 10.1016/j.biopsych.2005.01.003. PMID: 15780858

[13] Gold JM Goldberg RW McNary SW Dixon LB Lehman AF . Cognitive correlates of job tenure among patients with severe mental illness. Am J Psychiatry. (2002) 159:1395–402. doi: 10.1176/appi.ajp.159.8.1395. PMID: 12153834

[14] Ventura J Subotnik KL Gitlin MJ Gretchen-Doorly D Ered A Villa KF . Negative symptoms and functioning during the first year after a recent onset of schizophrenia and 8 years later. Schizophr Res. (2015) 161:407–13. doi: 10.1016/j.schres.2014.10.043. PMID: 25499044 PMC4308531

[15] Tripathi A Kar SK Shukla R . Cognitive deficits in schizophrenia: understanding the biological correlates and remediation strategies. Clin Psychopharmacol Neurosci. (2018) 16:7–17. doi: 10.9758/cpn.2018.16.1.7. PMID: 29397662 PMC5810454

[16] Butcher I Berry K Haddock G . Understanding individuals' subjective experiences of negative symptoms of schizophrenia: a qualitative study. Br J Clin Psychol. (2020) 59:319–34. doi: 10.1111/bjc.12248. PMID: 32242945

[17] Halverson TF Orleans-Pobee M Merritt C Sheeran P Fett AK Penn DL . Pathways to functional outcomes in schizophrenia spectrum disorders: meta-analysis of social cognitive and neurocognitive predictors. Neurosci Biobehav Rev. (2019) 105:212–9. doi: 10.1016/j.neubiorev.2019.07.020. PMID: 31415864

[18] Herkströter F Zahedi A Standke I Dannlowski U Lencer R Schubotz RI . Gray matter matters: cognitive stability and flexibility in schizophrenia spectrum disorder. Psychophysiology. (2024) 61:e14596. doi: 10.1111/psyp.14596 38691383

[19] Naim-Feil J Rubinson M Freche D Grinshpoon A Peled A Moses E . Altered brain network dynamics in schizophrenia: a cognitive electroencephalography study. Biol Psychiatry Cognit Neurosci Neuroimaging. (2018) 3:88–98. doi: 10.1016/j.bpsc.2017.03.017. PMID: 29397084

[20] Wang Y Ouyang L Fan L Zheng W Li Z Tang J . Functional and structural abnormalities of thalamus in individuals at early stage of schizophrenia. Schizophr Res. (2024) 271:292–9. doi: 10.1016/j.schres.2024.07.045. PMID: 39079406

[21] Vita A Gaebel W Mucci A Sachs G Erfurth A Barlati S . European Psychiatric Association guidance on assessment of cognitive impairment in schizophrenia. Eur Psychiatry. (2022) 65:e58. doi: 10.1192/j.eurpsy.2022.2316. PMID: 36059109 PMC9532219

[22] Vita A Barlati S Cavallaro R Mucci A Riva MA Rocca P . Definition, assessment and treatment of cognitive impairment associated with schizophrenia: expert opinion and practical recommendations. Front Psychiatry. (2024) 15:1451832. doi: 10.3389/fpsyt.2024.1451832. PMID: 39371908 PMC11450451

[23] Feber L Peter NL Chiocchia V Schneider-Thoma J Siafis S Bighelli I . Antipsychotic drugs and cognitive function: a systematic review and pairwise network meta-analysis. JAMA Psychiatry. (2024) 82:47–56. doi: 10.1186/s13643-023-02213-5. PMID: 39412783 PMC11581732

[24] Kaar SJ Natesan S McCutcheon R Howes OD . Antipsychotics: mechanisms underlying clinical response and side-effects and novel treatment approaches based on pathophysiology. Neuropharmacology. (2020) 172:107704. doi: 10.1016/j.neuropharm.2019.107704. PMID: 31299229

[25] Vita A Gaebel W Mucci A Sachs G Barlati S Giordano GM . European Psychiatric Association guidance on treatment of cognitive impairment in schizophrenia. Eur Psychiatry. (2022) 65:e57. doi: 10.1192/j.eurpsy.2022.2315. PMID: 36059103 PMC9532218

[26] Gouveia M Ascenção R Fiorentino F Pascoal J Costa J Borges M . The cost and burden of schizophrenia in Portugal in 2015. Int J Clin Neurosci Ment Health. (2017) 4(suppl.3):s13. Available online at: https://arc-publishing.org/IJCNMH-Sup_Material/IJCNMH.2017.4.Sp3.S13-Portuguese.pdf.

[27] Gattrell WT Logullo P van Zuuren EJ Price A Hughes EL Blazey P . ACCORD (ACcurate COnsensus Reporting Document): a reporting guideline for consensus methods in biomedicine developed via a modified Delphi. PloS Med. (2024) 21:e1004326. doi: 10.1371/journal.pmed.1004326. PMID: 38261576 PMC10805282

[28] Halcomb E Davidson P Hardaker L . Using the consensus development conference method in healthcare research. Nurse Res. (2008) 16:56–71. doi: 10.7748/nr2008.10.16.1.56.c6753. PMID: 19025106

[29] Oliveira J . The Brief Cognitive Assessment Tool for Schizophrenia: Exploratory study in a sample of Portuguese patients (2021). Available online at: https://estudogeral.uc.pt/bitstream/10316/98505/1/Tese%20final.pdf (Accessed March 21, 2026).

[30] Couto BO R Gonçalves AS Monteleone F Oliveira S Fonseca JB Rodrigues R . Screen for Cognitive Impairment in Psychiatry (SCIP): adaptation and validation for Portuguese version. Eur Psychiatry. (2024) 67:S160. doi: 10.1192/j.eurpsy.2024.356. PMID: 38385431

[31] Coentre R Levy P . Early intervention in psychosis: the first national survey in Portugal. Schizophr Res. (2020) 218:298–9. doi: 10.1016/j.schres.2020.03.019. PMID: 32192795

[32] Coentre R Mendes T Rebelo A Fonseca A Levy P . PROFIP: a Portuguese early intervention programme for first-episode psychosis in Lisbon. Early Interv Psychiatry. (2019) 13:1525–9. doi: 10.1111/eip.12852. PMID: 31264775

[33] Vita A Barlati S Ceraso A Nibbio G Ariu C Deste G . Effectiveness, core elements, and moderators of response of cognitive remediation for schizophrenia: a systematic review and meta-analysis of randomized clinical trials. JAMA Psychiatry. (2021) 78:848–58. doi: 10.2139/ssrn.3687361. PMID: 33877289 PMC8058696

[34] Prikken M Konings MJ Lei WU Begemann MJH Sommer IEC . The efficacy of computerized cognitive drill and practice training for patients with a schizophrenia-spectrum disorder: a meta-analysis. Schizophr Res. (2019) 204:368–74. doi: 10.1016/j.schres.2018.07.034. PMID: 30097278

[35] Justo-Henriques SI Pérez-Sáez E Carvalho JO Cavallo M Sargaço P . Home-based individualized cognitive stimulation (iCS) therapy in Portuguese psychiatric patients: a randomized controlled trial. Brain Sci. (2022) 12:1655. doi: 10.3390/brainsci12121655. PMID: 36552114 PMC9775072

[36] Moura BM Avila A Chendo I Frade P Barandas R Vian J . Facilitating the delivery of cognitive remediation in first-episode psychosis: pilot study of a home-delivered web-based intervention. J Nerv Ment Dis. (2019) 207:951–7. doi: 10.1097/nmd.0000000000001055. PMID: 31503184

[37] Firth J Stubbs B Rosenbaum S Vancampfort D Malchow B Schuch F . Aerobic exercise improves cognitive functioning in people with schizophrenia: a systematic review and meta-analysis. Schizophr Bull. (2017) 43:546–56. doi: 10.1093/schbul/sbw115. PMID: 27521348 PMC5464163

[38] Shimada T Ito S Makabe A Yamanushi A Takenaka A Kawano K . Aerobic exercise and cognitive functioning in schizophrenia: an updated systematic review and meta-analysis. Psychiatry Res. (2022) 314:114656. doi: 10.1016/j.psychres.2022.114656. PMID: 35659670

[39] Lak M Jafarpour A Shahrbaf MA Lak M Dolatshahi B . The effect of physical exercise on cognitive function in schizophrenia patients: a GRADE assessed systematic review and meta-analysis of controlled clinical trials. Schizophr Res. (2024) 271:81–90. doi: 10.1016/j.schres.2024.07.020. PMID: 39013348

[40] Solmi M Croatto G Piva G Rosson S Fusar-Poli P Rubio JM . Efficacy and acceptability of psychosocial interventions in schizophrenia: systematic overview and quality appraisal of the meta-analytic evidence. Mol Psychiatry. (2023) 28:354–68. doi: 10.1038/s41380-022-01727-z. PMID: 35999275

[41] Patterson TL Leeuwenkamp OR . Adjunctive psychosocial therapies for the treatment of schizophrenia. Schizophr Res. (2008) 100:108–19. doi: 10.1016/j.schres.2007.12.468. PMID: 18226500

[42] Chien WT Leung SF Yeung FK Wong WK . Current approaches to treatments for schizophrenia spectrum disorders, part II: psychosocial interventions and patient-focused perspectives in psychiatric care. Neuropsychiatr Dis Treat. (2013) 9:1463–81. doi: 10.2147/ndt.s49263. PMID: 24109184 PMC3792827

[43] Simões do Couto F Queiroz C Barbosa T Ferreira L Firmino H Viseu M . Clinical and therapeutic characterization of a Portuguese sample of patients with schizophrenia. Actas Esp Psiquiatr. (2011) 39:147–54. Available online at: https://actaspsiquiatria.es/index.php/actas/article/view/615. 21560074

[44] Sinkeviciute I Begemann M Prikken M Oranje B Johnsen E Lei WU . Efficacy of different types of cognitive enhancers for patients with schizophrenia: a meta-analysis. NPJ Schizophr. (2018) 4:22. doi: 10.1038/s41537-018-0064-6. PMID: 30361502 PMC6202388

[45] Yolland COB Phillipou A Castle DJ Neill E Hughes ME Galletly C . Improvement of cognitive function in schizophrenia with N-acetylcysteine: a theoretical review. Nutr Neurosci. (2020) 23:139–48. doi: 10.1080/1028415x.2018.1478766. PMID: 29847303

[46] Singh A Kumar V Pathak H Jacob AA Venkatasubramanian G Varambally S . Effect of antipsychotic dose reduction on cognitive function in schizophrenia. Psychiatry Res. (2022) 308:114383. doi: 10.1016/j.psychres.2021.114383. PMID: 34999291

[47] Zhou Y Li G Li D Cui H Ning Y . Dose reduction of risperidone and olanzapine can improve cognitive function and negative symptoms in stable schizophrenic patients: a single-blinded, 52-week, randomized controlled study. J Psychopharmacol. (2018) 32:524–32. doi: 10.1177/0269881118756062. PMID: 29493377

[48] Haddad C Salameh P Sacre H Clément JP Calvet B . Effects of antipsychotic and anticholinergic medications on cognition in chronic patients with schizophrenia. BMC Psychiatry. (2023) 23:61. doi: 10.1186/s12888-023-04552-y. PMID: 36694187 PMC9872384

[49] Mancini V Latreche C Fanshawe JB Varvari I Zauchenberger CZ McGinn N . Anticholinergic burden and cognitive function in psychosis: a systematic review and meta-analysis. Am J Psychiatry. (2025) 182:349–59. doi: 10.1176/appi.ajp.20240260. PMID: 40007252

[50] Lystad JU Falkum E Mohn C Haaland V Bull H Evensen S . The MATRICS consensus cognitive battery (MCCB): performance and functional correlates. Psychiatry Res. (2014) 220:1094–101. doi: 10.1016/j.psychres.2014.08.060. PMID: 25242432

[51] Cowman M Lonergan E Burke T Bowie CR Corvin A Morris DW . Evidence supporting the use of a brief cognitive assessment in routine clinical assessment for psychosis. Schizophr (Heidelb). (2022) 8:113. doi: 10.1038/s41537-022-00322-z. PMID: 36528607 PMC9759520

[52] Keefe RS Goldberg TE Harvey PD Gold JM Poe MP Coughenour L . The brief assessment of cognition in schizophrenia: reliability, sensitivity, and comparison with a standard neurocognitive battery. Schizophr Res. (2004) 68:283–97. doi: 10.1016/j.schres.2003.09.011. PMID: 15099610

[53] Purdon SE . The screen for cognitive impairment in psychiatry (SCIP): instructions and three alternative forms. In: PNL inc, edmonton Alberta. (2005).

[54] Hurford IM Ventura J Marder SR Reise SP Bilder RM . A 10-minute measure of global cognition: validation of the brief cognitive assessment tool for schizophrenia (B-CATS). Schizophr Res. (2018) 195:327–33. doi: 10.1016/j.schres.2017.08.033. PMID: 28918221

[55] Amado I Sederer LI . Implementing cognitive remediation programs in France: the "Secret Sauce. Psychiatr Serv. (2016) 67:707–9. doi: 10.1176/appi.ps.201600033. PMID: 26975526

[56] Wykes T Huddy V Cellard C McGurk SR Czobor P . A meta-analysis of cognitive remediation for schizophrenia: methodology and effect sizes. Am J Psychiatry. (2011) 168:472–85. doi: 10.1176/appi.ajp.2010.10060855. PMID: 21406461

[57] Keepers GA Fochtmann LJ Anzia JM Benjamin S Lyness JM Mojtabai R . The American Psychiatric Association practice guideline for the treatment of patients with schizophrenia. Am J Psychiatry. (2020) 177:868–72. doi: 10.1176/appi.ajp.2020.177901. PMID: 32867516

[58] Suetani S Dark F Every-Palmer S Galletly C O’Donoghue B Halstead S . Australian and New Zealand Journal of Psychiatry grading of recommendations, assessment, development and evaluations (GRADE) guidelines for the management of schizophrenia. Aust New Z J Psychiatry. 60:367–404. doi: 10.1177/00048674251406058. PMID: 41540779 PMC13000005

[59] VA/DoD Clinical Practice Guideline Management of First-Episode Psychosis and Schizophrenia Work Group . Management of first-episode psychosis and schizophrenia work group. Washington, DC: U.S. Government Printing Office (2023). Available online at: https://www.healthquality.va.gov/guidelines/MH/scz/index.asp (Accessed March 21, 2026).

[60] Øie MG Barbosa F Lettner S Baker G Duarte C Hessen E . Why clinical neuropsychology matters in schizophrenia care. Front Psychol. (2025) 16:1713751. doi: 10.3389/fpsyg.2025.1713751. PMID: 41426408 PMC12715811

[61] Waggoner J Carline JD Durning SJ . Is there a consensus on consensus methodology? Descriptions and recommendations for future consensus research. Acad Med. (2016) 91:663–8. doi: 10.1097/acm.0000000000001092. PMID: 26796090

[62] Schünemann HJ Brennan S Akl EA Hultcrantz M Alonso-Coello P Xia J . The development methods of official GRADE articles and requirements for claiming the use of GRADE – a statement by the GRADE guidance group. J Clin Epidemiol. (2023) 159:79–84. doi: 10.1016/j.jclinepi.2023.05.010. PMID: 37211327

